# Effects of short video addiction on college students’ physical activity: the chain mediating role of self-efficacy and procrastination

**DOI:** 10.3389/fpsyg.2024.1429963

**Published:** 2024-10-31

**Authors:** Zhe Zhao, Yali Kou

**Affiliations:** ^1^Department of Physical Education, Kunsan National University, Gunsan, Republic of Korea; ^2^School of Marxism, Shangqiu Normal University, Shangqiu, China

**Keywords:** short video addiction, physical activity, self-efficacy, procrastination, chain mediating

## Abstract

**Introduction:**

Excessive use of short video applications can adversely affect the physical and mental health of college students. At present, regarding the effect of short video addiction on physical exercise, few scholars have studied the mechanism of action. This study aims to investigate the mechanism by which short video addiction impacts college students’ physical exercise. Therefore, we investigated the correlation between short video addiction and physical activity, and examined the influence of self-efficacy and procrastination on this relationship.

**Methods:**

In this research, 304 college students were selected as survey subjects. The questionnaires included Short Video Addiction Scale, Physical Activity Rating Scale, General Self-Efficacy Scale, and Short Version General Procrastination Scale. The data underwent correlation analysis using SPSS and mediation effect analysis using the PROCESS macro program.

**Results:**

(1) 61.51% (187) of college students’ physical activity was low exercise. (2) Physical activity was significantly negatively impacted by short video addiction. (3) Self-efficacy played an independent mediating role in the association between short video addiction and physical activity. (4) The association between short video addiction and physical activity was independently mediated by procrastination. (5) Self-efficacy and procrastination function as chain mediators in the association between short video addiction and physical activity.

**Discussion:**

Our research identifies the role that self-efficacy and procrastination play in the connection between short video addiction and physical activity. Decreasing the utilization of short video applications and enhancing self-efficacy can reduce procrastination and improve physical activity for college student groups.

## Introduction

Currently, short video applications are famous worldwide and have gradually become the most popular mobile applications college students use. Short video apps have enriched college students’ daily lives and entertainment, but the phenomenon of short video addiction has become increasingly severe among the college student population. Short video apps’ ease of use and entertainment features reinforce the tendency toward overuse. In a survey of 1,345 adolescents on TikTok short-video app use, 461 addicted users were found, and addicted users showed poorer mental health than non-users (Miao et al., 2023). College students’ physical and emotional health may be impacted by short video addiction, such as sedentary behavior, sleep disorders, and decreased academic performance (Kwok et al., 2021). Despite detrimental impacts, young people use short video apps often. The video feature of short video apps activates users’ satisfaction and withdrawal, which has implications for addiction (Tian et al., 2023). Facing heavy academic and employment pressure, short video applications have become one of the essential ways for college students to relieve pressure and communicate with the outside world. College students are among the most sedentary groups, and the sedentary time associated with entertainment screens accounts for about 35% of the total sedentary time (Carpenter et al., 2021). Lepp et al. found that excessive smartphone use interferes with physical activity (Lepp et al., 2013). Previous research shows that participation in physical activity is an effective way to improve people’s smartphone addiction problems (Liu et al., 2019). College students’ physical and cognitive health improves with regular physical activity. Research on the intrinsic mechanisms and mediating roles of short video addiction and physical activity has not been completely revealed. This study aims to fill this research void. It is significant for solving the problem of short video addiction and encouraging them to participate more in physical exercise. This study expands theoretical research on physical activity influencing factors and gives educators and college students practical experience to reduce short video addiction and physical activity.

### Short video addiction and physical activity

Short video apps have brought convenience to people’s lives but also affect physical activity. Excessive use of short video apps can lead to addiction. Short video addiction is the extent to which users develop a solid attachment to short videos and uncontrollably reuse them, resulting in general addiction symptoms (Mu et al., 2022). Short video apps’ social communication and video entertainment features are closely related to smartphone addiction. Research has identified a high and significant positive link between smartphone addiction and social media use (Arnavut et al., 2018). Marengo et al. found that duration of smartphone use and use of TikTok were the strongest predictors of social media addiction (Marengo et al., 2022). Therefore, short video addiction is more like a new manifestation of smartphone addiction that has developed to a particular stage. Physical exercise is one of the essential means to promote people’s physical health. While smartphones bring convenience to people’s lives, they also affect the way people are physically active. Prior research has connected problematic smartphone use to inactivity and sedentary lifestyles (Fennell et al., 2019). Smartphone-addicted participants were less likely to walk daily (Xiang et al., 2020). The adverse effects of smartphone addiction on physical activity can be found. It has been found that increased physical activity among adolescents can reduce short video addiction behavior to some extent (He et al., 2024). Therefore, we propose Hypothesis 1: Short video addiction has a significant negative predictive effect on physical activity.

### The mediating role of self-efficacy

Psychosocial factors influencing physical activity are usually related to self-efficacy. Self-efficacy is a person’s beliefs about his or her ability to perform the actions necessary to meet situational demands (Bandura, 1986). Theoretically, self-efficacy influences people’s choices of activities, the amount of effort they put into them, and how persistent they are when faced with negative stimuli (McAuley et al., 1994). Psychosocial factors influencing physical activity most often involve self-efficacy. Increasing levels of self-efficacy can help people initiate and maintain a regular physical activity program (Pekmezi et al., 2009). Young et al. showed a stable positive link between self-efficacy and physical activity (Young et al., 2014). Researchers in the field of addictive behaviors have long used the concept of self-efficacy in theory and research. Self-efficacy measures have been useful in assessing and treating addictions like smoking and alcoholism (DiClemente et al., 1995). Additionally, related research has discovered a negative correlation between smartphone addiction and self-efficacy (Kim and Jung, 2015). Self-efficacy can mitigate the negative effects associated with smartphone addiction (Cheng et al., 2021). Smartphone addiction might lower self-efficacy and prevent physical activity (Lin et al., 2022). An analysis of short video addiction revealed a negative effect on creative self-efficacy (Lin et al., 2023). Based on the above, it may be inferred that addiction to short videos lowers self-efficacy, which in turn lowers physical activity participation. This study proposes Hypothesis 2: Self-efficacy mediates the association between short video addiction and physical activity.

### The mediating role of procrastination

Research on procrastination is relatively well established. Procrastination is “voluntarily postponing a scheduled action, knowing that the delay will worsen the situation” (Steel, 2007). Procrastination leads to college students’ academic and personal struggles. Some studies have reported that about 70–80% of college students have procrastination behaviors of different degrees (Geng et al., 2018). With the development of intelligent electronic devices, more and more college students are addicted to them, and their procrastination behavior is getting more serious. Characteristic procrastination positively correlates with college students’ cell phone addiction (Yang et al., 2020). College students will exhibit avoidance behavior in the face of study pressure, and addiction to short videos will harm learners’ motivation and positive learning psychology (Ye et al., 2022). Academic procrastination has a significant negative effect on physical activity among college students (Ren et al., 2021). In a survey of 610 college students, Tao et al. found a strong negative association between procrastination and physical activity (Tao et al., 2022). Given the effect of procrastination on the connection between short video addiction and physical activity, this study proposes Hypothesis 3: Procrastination mediates the association between short video addiction and physical activity.

### The chain mediating role of self-efficacy and procrastination

Self-efficacy is an essential factor in studying procrastination behavior. According to earlier research, procrastination is significantly and negatively predicted by self-efficacy (Haycock, 1993; Klassen et al., 2008). Low self-efficacy students are more likely to get stuck in a procrastination loop (Wäschle et al., 2014). Low self-control, low self-efficacy, and sleeping late are positive predictors of procrastination (Przepiórka et al., 2019). Li et al. showed that academic self-efficacy buffered smartphone addiction and academic procrastination (Li et al., 2020). Physical inactivity and mobile phone addiction increased illogical procrastination (Shi et al., 2021.). In conclusion, considering the connection and function of self-efficacy and procrastination, improving college students’ self-efficacy is conducive to reducing procrastination behavior, which may lessen the detrimental effects of short video addiction on physical activity. Therefore, this study proposes Hypothesis 4: Self-efficacy and procrastination play a chain mediating role between short video addiction and physical activity.

## Materials and methods

### Participants

This study was conducted at two universities in Henan Province, China. Participants in this survey were recruited voluntarily. Because the study did not obtain enough volunteers at the first university at the beginning of the study, a second university was chosen to continue recruiting volunteers. In order to minimize the discrepancy between the profiles and results of college students at these two schools, both universities were chosen to be in Henan Province. They were Normal Universities with similar profiles of enrolled students. Participants were full-time undergraduates from first-year students through seniors at both universities. For the diversity and representativeness of the sample, the samples were stratified and randomly selected based on the student’s gender and grade level.

Several faculty members from both universities were invited to serve as test administrators for distributing and collecting the questionnaires. The questionnaire was filled out through the Questionnaire Star website. The questionnaire and scale filling instructions were given to responders at the start of the test. Data confidentiality, voluntary completion, and anonymity were stressed in the test. All test subjects confirmed informed consent to participate and could withdraw from the test at any time. In order to guarantee the validity of the data, questionnaires that were answered too quickly, filled out with the regularity of answers, and filled out invalid questionnaires were excluded. From 320 questionnaires, 304 were valid after recovering and eliminating invalid ones, a 95% recovery rate. The age of the respondents was 20.94 ± 1.68 years old, of which 113 (37.2%) were male and 191 (62.8%) were female. There were 94 (30.9%) in grade one, 93 (30.6%) in grade two, 84 (27.6%) in grade three, and 33 (10.9%) in grade four.

### Measures

#### Short video addiction scale

The Short Video Addiction Scale is stable, dependable, and can measure college students’ short video addiction individually (Qin, 2020). The scale includes four dimensions: withdrawal, avoidance, loss of control, and ineffectiveness. The scale consists of 14 items, each rated on a scale of 1–5 (very inconsistent ~ very consistent). The scale has 14 items, including withdrawal, avoidance, loss of control, and ineffectiveness. The four factors have excellent structural fit and a 0.91 total scale internal consistency reliability coefficient. Cronbach’s *α* was 0.89 in this study.

#### Physical activity rating scale

The physical activity rating scale (PARS-3), revised by Liang (1994). Three factors are considered in the scale: time, intensity, and frequency. Exercise amount = intensity×(time−1) × frequency. Intensity, frequency and time are scored from 1 to 5 on a scale of 1–5. Scale scores varied from 0 to 100. Exercise amounts were assessed as follows: ≤19 for low, 20–42 for moderate, and ≥43 for high. The PARS-3’s retest reliability was 0.82. In this study, Cronbach’s α was 0.71.

#### General self-efficacy scale

The Chinese version of the GSES was translated and revised by Zhang and Schwarzer (1995). The scale has one dimension and consists of 10 items scored on a 4-point Likert scale. The overall score on the scale spans from 10 to 40. Higher scores indicate greater self-efficacy. The internal consistency coefficient was 0.87. Cronbach’s *α* was 0.93, in this research.

#### Short general procrastination scale

The SGPS was scaled down from the General Delay Scale by Sirois et al. (2019). The Chinese version of the SGPS was translated and revised by Zhang et al. (2020). The SGPS consists of 9 questions, of which 3 questions are reverse scored. The questions are scored on a 5-point Likert scale (very inconsistent ~ very consistent). Higher total scores imply a stronger tendency to procrastinate. The internal consistency reliability of the SGPS is 0.87. Cronbach’s α was 0.77, in this research.

### Statistical analyses

SPSS 26.0 was used to conduct the statistical analysis. First, the data were obtained through a questionnaire. To check for common method bias, we employed the Harman one-way test. Eight factors had a characteristic root larger than 1, and the first factor explained 24.65% variation, indicating no significant common method bias. Then, participants’ individual characteristics were analyzed descriptively, including means and standard deviations. Correlations were computed using Spearman’s correlation analysis. Finally, Hayes’ PROCESS macro in SPSS was used for mediation analysis. The data was bootstrapped with 5,000 samples to obtain 95% confidence intervals (CI) (Shrout and Bolger, 2002). The mediating effect was considered significant if the upper and lower values of the 95% confidence interval did not contain 0 between them.

## Results

Based on the criterion of low exercise level with a total physical activity score ≤ 19, the detection rate of low exercise level in physical activity was 61.51% (187). Table 1 shows each variable’s descriptive statistics and correlation coefficients. Following Pearson’s correlation analysis, the college students’ short-video addiction was negatively linked with self-efficacy (*r* = −0.187, *p* < 0.01), significantly positively associated with procrastination (*r* = 0.397, *p* < 0.01), and significantly negatively associated with physical activity (*r* = −0.259, *p* < 0.01). Self-efficacy was significantly negatively associated with procrastination (*r* = −0.311, *p* < 0.01) and positively associated with physical activity (*r* = 0.313, *p* < 0.01). Procrastination was significantly negatively associated with physical activity (*r* = −0.281, *p* < 0.01).

**Table 1:** Correlations analysis between the variables.

	*M*	SD	Short video addiction	Physical activity	Self-efficacy	Procrastination
Short video addiction	40.29	10.25	1	–	–	–
Physical activity	21.19	20.71	−0.259^**^	1	–	–
Self-efficacy	23.58	5.87	−0.187^**^	0.313^**^	1	–
Procrastination	25.06	5.43	0.397^**^	−0.281^**^	−0.311^**^	1

*n* = 304, **p < 0.01.

SPSS 26.0 and Hayes’ SPSS macro tool administered a bootstrap-based mediation effects test using Model 6 (Hayes, 2013). The mediation effects were tested by controlling for gender, age, and grade level. The results of the regression analysis (Table 2) showed that short video addiction was a significant negative predictor of physical activity (*β* = −0.252, *p* < 0.001), a significant negative predictor of self-efficacy (β = −0.186, *p* < 0.01), and a significant positive predictor of procrastination (β = 0.343, *p* < 0.001). Procrastination was significantly predicted negatively by self-efficacy (β = −0.289, *p* < 0.001). After adding self-efficacy and procrastination to the regression equation, physical activity was considerably positively predicted by self-efficacy (β = 0.202, *p* < 0.001). Procrastination significantly negatively predicted physical activity (β = −0.192, *p* < 0.01). Short video addiction still negatively predicted physical activity (β = −0.138, *p* < 0.05).

**Table 2:** Regression analysis between the variables.

Regression equation		Overall fit index			Significance of regression coefficient	
Result variable	Predictive variable	*R*	*R*2	*F*	β	*t*
Physical activity	Gender	0.313	0.098	8.134	−0.155	−2.795^**^
	Age				−0.034	−0.394
	Grade				−0.056	−0.654
	Short video addiction				−0.252	−4.578^***^
Self-efficacy	Gender	0.248	0.061	4.891	−0.150	−2.659^**^
	Age				−0.128	−1.455
	Grade				0.111	1.257
	Short video addiction				−0.186	−3.314 ^**^
Procrastination	Gender	0.539	0.290	24.387	−0.265	−5.333^***^
	Age				−0.115	−1.489
	Grade				0.168	2.182
	Short video addiction				0.343	6.882 ^***^
	Self-efficacy				−0.289	−5.747^***^
Physical activity	Gender	0.432	0.187	11.376	−0.167	−2.990^**^
	Age				−0.023	−0.277
	Grade				−0.053	−0.635
	Short video addiction				−0.138	−2.402^*^
	Self-efficacy				0.202	3.556^***^
	Procrastination				−0.192	−3.095^**^

*p < 0.05, **p < 0.01, ***p < 0.001.

**Figure 1:**
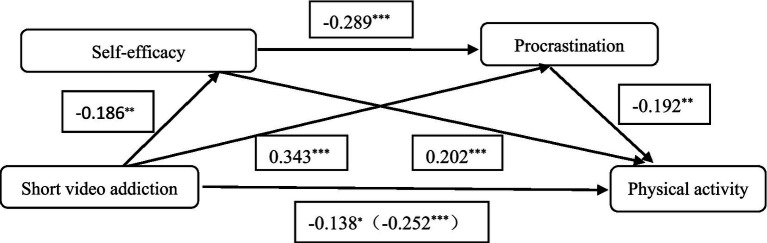
Model of mediating roles of self-efficacy and procrastination between short video addiction and physical activity. *p < 0.05, **p < 0.01, ***p < 0.001.

The mediation analysis (Table 3; Figure 1) showed that the total mediation effect value was −0.235, accounting for 45.11% of the total effect of brief video addiction on physical activity (effect value −0.521). Three pathways constitute this mediating effect: first, short video addiction → self-efficacy → physical activity, with a mediating effect value of −0.078 (14.97%); second, short video addiction → procrastination → physical activity, with a mediating effect value of −0.136 (26.11%). Third, short video addiction → self-efficacy → procrastination → physical activity, with a mediated effect value of −0.021 (4.03%).

**Table 3:** Mediation effect test based on bootstrap.

Benefit type	Effect value	BootSE	Bootstrap 95% CI		Proportion of relative effect
			Boot LLCI	Boot ULCI	
Direct effect	−0.286	0.119	−0.519	−0.052	54.89%
Indirect effect 1	−0.078	0.041	−0.172	−0.012	14.97%
Indirect effect 2	−0.136	0.059	−0.265	−0.036	26.11%
Indirect effect 3	−0.021	0.014	−0.054	−0.002	4.03%
Total indirect effect	−0.235	0.081	−0.406	−0.092	45.11%
Total effect	−0.521	0.114	−0.745	−0.297	

## Discussion

In this study, we examined short video addiction and physical activity. Results showed that 61.51% (187) of college students’ physical activity was low (score ≤ 19). This indicates a high percentage of college students with low physical activity, as confirmed by previous studies (Castro et al., 2020). In the pre-smartphone era, researchers found that about 40–50 percent of college students were physically inactive (Keating et al., 2005). During the same period, studies of physical activity among college students of different races and countries have also demonstrated physical inactivity among college students (Suminski et al., 2002; Irwin, 2004). In summary, college students’ physical activity levels have been chronically low. A report on the state of China’s Internet industry pointed out that as of 2023, China’s mobile Internet user’s *per capita* single-day use time is 435 min, of which short video *per capita* single-day use time is 151 min (China Netcasting Services Association, 2024). College students are among the primary users of the Internet. With the development of short video apps, the time spent by college students on short video apps gradually increases and takes up most of their time using the Internet (Zhang et al., 2023). Overuse behaviors are more likely to occur in college years. This study found that short-video addiction had a significant negative effect on physical activity, which supports prior studies on short-video addiction and physical activity (Ali and Ali, 2023). Research hypothesis 1 was validated. It indicates that short video addiction has a negative effect on the physical activity behavior of college students. Long-term addiction to short video applications is detrimental to participation in physical activity. Therefore, reducing the usage of short video apps is beneficial to enhancing the physical activity level of college students.

The study’s results suggest that short video addiction affects physical activity through the independent mediating role of self-efficacy. Research hypothesis 2 was verified. College students who are addicted to short videos will have lower levels of self-efficacy, which will impact their level of physical activity. As an important concept in social cognitive theory, self-efficacy influences individual behavior, motivation, and psychological state. Self-efficacy has been used as a mediating or moderating variable that influences the relationship between smartphone addiction and psychological factors such as anxiety, stress, and loneliness (Li et al., 2021; Chiu, 2014; Cheng et al., 2021). According to Bandura, individual behavior is formed by interacting with personal factors and the external environment (Bandura, 1999). When college students indulge in the online virtual environment created by short videos for an extended period, individual self-efficacy decreases, and the interaction of the natural social environment is weakened, reducing physical activity behavior. Therefore, self-efficacy is an essential factor affecting physical activity for college students addicted to short videos. Reducing short video apps and enhancing self-efficacy can boost physical activity.

It was found that short video addiction affects physical activity through the separate mediating role of procrastination. Research hypothesis 3 was tested. Chronic use of short videos by college students increases procrastination behavior. College students who are addicted to smartphones will procrastinate more when completing their studies (Kaya, 2024). College students need to face various pressures such as academics, employment, and life, and when they are unable to alleviate these pressures reasonably, procrastination behaviors will occur. Procrastination may substitute healthy behaviors like exercise and sleep as stress rises (Åsberg and Bendtsen, 2023). Highly illogical procrastination is positively associated with a lack of exercise and smartphone addiction (Shi et al., 2021). For college students addicted to short videos, procrastination is a negative factor that prevents them from actively participating in physical activity. The neurophysiological mechanisms of short-video addictive behaviors may impair brain regions associated with self-regulation, thereby reducing an individual’s inhibitory control. Therefore, reducing short-video use is crucial to helping students mitigate the effects of procrastination and actively participate in physical activity.

Our results imply that short video addiction influences physical activity through a chain mediation effect of self-efficacy and procrastination. Research hypothesis 4 was tested. In particular, self-efficacy is a significant negative predictor of procrastination; the degree of procrastination decreases with increasing self-efficacy. This aligns with earlier studies (Ariani and Susilo, 2018). The knock-on mediating effect suggests that college students’ addiction to short videos negatively predicts their self-efficacy, which affects procrastination. Procrastination can affect an individual’s positive motivation and even behavior (Kelly and Walton, 2021). Procrastination can negatively affect an individual’s development, which is evident in physical activity. Research findings show a significant negative predictive effect between procrastination and physical activity; the higher the level of procrastination. Self-efficacy significantly mediated the association between physical activity and academic procrastination (Ren et al., 2021). When a college student’s self-efficacy is lowered, he may engage in procrastination behaviors that reduce the likelihood of participating in physical activity. The higher the addiction to short videos, the greater the impact of self-efficacy and procrastination behaviors on physical activity. College students addicted to short videos were likelier to experience decreased self-efficacy, increased procrastination behaviors, and decreased physical activity participation. Baumeister et al.’s ego depletion hypothesis states that (Baumeister et al., 1998), an individual’s ego activity leads to the depletion of psychological energy, which results in declining executive function. This theory believes that human psychological energy is limited, and self-regulation requires energy; when energy is exhausted, self-regulation will decline, self-control will also decline, and procrastination will be easier. Therefore, when college students use short video apps for a long time, they overly consume their own psychological energy, which results in the depletion of self-control, decreasing self-efficacy and procrastination behaviors, and affecting the level of physical activity. Our study shows that college students reduce the excessive use of short videos, increase self-efficacy, reduce procrastination behaviors, and improve physical activity levels. Previous research has indicated that smartphone video addiction is closely related to physical activity. However, there needs to be more discussion on the correlation between short-video addiction and physical activity. The present study is valuable in answering this question from a media psychology perspective.

In the current digital era, new technologies represented by short videos are influencing college students’ physical activity. Research on the factors affecting college students’ physical activity can provide us with new ways to change their physical activity. The impact of short video addiction on physical activity reveals a new prevention and intervention model that emphasizes the importance of enhancing self-efficacy and reducing procrastination behaviors. In further research, we will focus on developing interventions to ameliorate the effects of short-video addiction on physical activity. This will reduce the adverse effects of short video addiction and increase physical activity levels among college students.

### Limitation and prospect

The study’s results are useful theoretically and as practical guidelines, but there are certain restrictions. First, the study was cross-sectional and collected data once, and there was no long-term continuous research study to determine causal relationships. Future studies could use longitudinal follow-up or experimental intervention design studies to determine the causal association between these factors. Second, this study only examined short video addiction, which affects physical activity, and the chain mediation model established is not the only mediation model due to the wide number of factors affecting the explanatory variables. Others may mediate, like self-regulation and anxiety. Thus, further research is needed to determine how these variables affect physical activity and short video addiction. We can conduct more research on the long-term impacts of short video addiction on physical activity and use self-efficacy theory to design interventions to reduce the detrimental effects of short video addiction on physical activity.

## Conclusion

The study found that short video addiction negatively predicts physical activity. Self-efficacy and procrastination are independent mediators of the relationship between short video addiction and physical activity. Short video addiction can affect physical activity through self-efficacy and procrastination, respectively, or through a third pathway, the chain-mediated effect of self-efficacy and procrastination. The preceding data imply that short video addiction directly affects physical activity levels and that self-efficacy and procrastination affect the relationship. Therefore, mental health lectures and counseling activities are organized for college students to help them improve self-efficacy and reduce procrastination behaviors, which is conducive to reducing the negative impact of short video addiction on physical activity.

## Data Availability

The original contributions presented in the study are included in the article/supplementary material, further inquiries can be directed to the corresponding author.
